# Models for Predicting the Adult Height and Age at First Menstruation of Girls with Idiopathic Central Precocious Puberty

**DOI:** 10.1371/journal.pone.0120588

**Published:** 2015-04-02

**Authors:** Eloïse Giabicani, Pierre Lemaire, Raja Brauner

**Affiliations:** 1 Fondation Ophtalmologique Adolphe de Rothschild, Paris, France; 2 Université Grenoble Alpes and CNRS, G-SCOP, F-38000 Grenoble, France; 3 Université Paris Descartes and Fondation Ophtalmologique Adolphe de Rothschild, Paris, France; John Hopkins University School of Medicine, UNITED STATES

## Abstract

**Background:**

It is difficult to determine whether to treat a given girl who has idiopathic central precocious puberty (CPP) with gonadotropin-releasing hormone analog (GnRHa) in terms of adult height (AH). The objective was to provide an easy tool for predicting AH and age at first menstruation at initial evaluation to help guide the decision regarding whether to treat.

**Methods:**

Data analysis using multiple linear regression models was performed in 134 girls with CPP. Among them 78 were given GnRHa because of low predicted AH (n=45), pubertal luteinising hormone (LH)/follicle-stimulating hormone peaks (FSH) ratio (n=50) and/or high plasma estradiol concentration (n=45). 56 girls were followed without treatment.

**Results:**

In the whole population, the actual AH (162.1±5.61 cm) was similar to target height (161.7±4.91 cm) and to AH predicted by the Bayley and Pinneau method (161.9±7.98 cm). Separated models for treated and untreated girls provide very close estimations, leading to a unique formula for both groups. The AH (cm) could be calculated at the initial evaluation: 2.21 (height at initial evaluation, SD) + 2.32 (target height, SD) – 1.83 (LH/FSH peaks ratio) + 159.68. The actual AH was lower than the calculated AH by more than 1 SD (5.6 cm) in 11 girls (8.0%). The time between onset of puberty and first menstruation (in untreated girls) can be estimated with: 10.9 - 0.57 (LH/FSH peaks ratio). The formulae are available at http://www.kamick.org/lemaire/med/girls-cpp15.html.

**Conclusions:**

We established formulae that can be used at an initial evaluation to predict the AH, and the time between onset of puberty and first menstruation after spontaneous puberty. The similarity of the formulae for both groups suggests that the treatment had no significant effect on the AH. However, the criteria used to select treatment suggest that it prevents the deterioration of AH in cases with rapidly evolving form of CPP.

## Introduction

Central precocious puberty (CPP) in girls is defined as the development of sexual characteristics before the age of 8 years due to the premature activation of the hypothalamo-pituitary-ovarian axis [1]. In girls, CPP is idiopathic in the majority of cases.

The premature secretion of estradiol increases the growth rate and accelerates bone maturation, which can shorten the growing period, resulting in short adult height (AH). Treatment with gonadotropin-releasing hormone (GnRH) analog (GnRHa) blocks the pituitary-ovarian axis and thus the secretion of estradiol, thereby slowing bone age (BA) progression and preserving growth potential [2]. However, the effect of this treatment on AH fluctuates, primarily because the progression of idiopathic CPP varies between unsustained forms, also called slowly progressing forms [3], and rapidly progressing forms [4]. Because of this variability, randomized prospective trials are not appropriate means for deciding treatment.

Despite the breadth of reported data concerning AH in girls with idiopathic CPP, major questions remain among the indications of GnRHa treatment [5]. The reported height gain (AH-predicted AH at onset of treatment) varies from 0.3 [6] to 9.8 cm [7]. Therefore, it is difficult to determine whether to treat a given girl who has idiopathic CPP with GnRHa. The Consensus Conference Group has recommended that progressive pubertal development be documented for 3–6 months before starting GnRHa treatment and that the responses to the GnRH test and estradiol assays should be monitored [1]. The factors reported to be associated with better results of GnRHa treatment for AH are: a younger chronological age at puberty [8,9] or treatment [7–10], a shorter interval between the onset of CPP and treatment [7], and greater height at initial evaluation [8,9,11,12], target height (TH) [9,12], predicted AH before treatment [8,12], duration of the treatment [7,10,11] and BA or chronological age at the end of treatment [7,11].

In a previous study [13], we identified models for predicting the difference between the AH and TH of girls with idiopathic CPP treated with GnRHa (n = 70) or untreated (n = 52), and the time between puberty onset and first menstruation. In the present study, we simplified these models by predicting AH (and not its difference with TH) and added 12 additional recent patients. Our objective was to provide an easy tool for predicting AH and age at first menstruation to help guide the decision regarding whether to treat with GnRHa or not.

## Materials and Methods

### Ethics statement

Written informed consent for the evaluations was obtained from the children’s parents and included in their hospital medical record. All clinical investigations were conducted according to the principals expressed in the Declaration of Helsinki. The Ethical Review Committee (Comité de Protection des Personnes, Ile de France III) approved this retrospective study and stated that ‘‘This study appears to be in accordance with the scientific principles generally accepted and to the ethical standards of research. The study was lead in the respect of the French law and regulation”. Patients information was anonymized prior to analysis.

### Patients

This retrospective, single-center study was carried out on 134 girls who were first seen for idiopathic CPP by a senior pediatric endocrinologist (R Brauner) between 1981 and 2007 (over 26 years) in a university pediatric hospital; this study includes the cohorts previously reported on AH [2,13,14]. The girls were older than 14 years of age (born before 1999) and had reached their AH (growth during the preceding year of less than 1 cm in a menstruating girl). CPP was diagnosed according to the appearance of breast development before the age of 8 years, accompanied by the presence of pubic or axillary hair (n = 86), a growth rate greater than 2 SD scores (SDS) the year before initial evaluation (n = 63) and/or a BA more than 2 years greater than their chronological age (n = 50) [15]. Organic intracranial lesions were excluded by neuroradiological evaluation in all but 6 (3.1%) cases who had a familial history of early puberty, normal neurological evaluation, were older than 6 years at the onset of puberty and had low plasma estradiol concentrations (<15 pg/mL) [16]. Ovarian [17], thyroid and adrenal disorders [18] were also excluded among girls with hair development as the first sign, as were those who were overweight.

**Fig 1:**
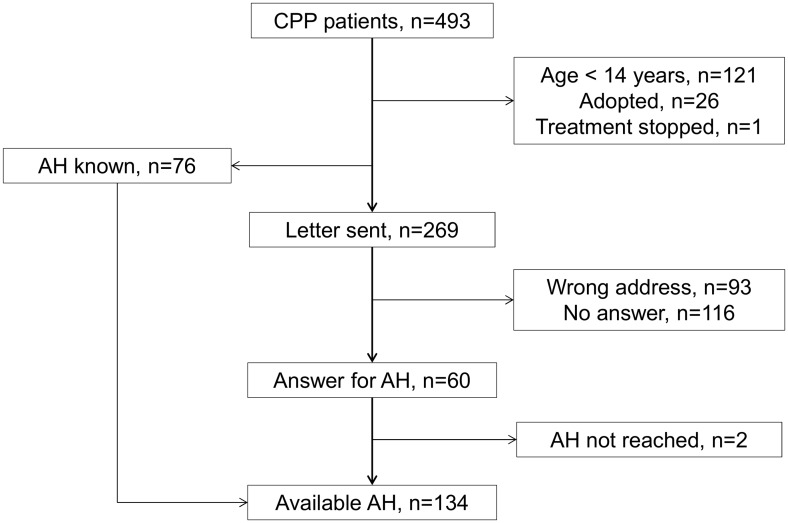
Flow chart of the inclusions of girls with CPP.

The 134 girls were selected from 493 girls with idiopathic CPP [15] (Fig. 1). The age at first menstruation was known for 129 of them. At the initial evaluation, the characteristics of the 134 girls included in the study were similar to those of the 359 girls without AH available, except for the BA advance, BMI, TH and plasma estradiol concentrations (Table 1).

**Table 1:** Comparison of the characteristics of included and excluded patients.

			Patients included			Patients excluded			MWUt
		Remarkable values	n	mean ± SD	%	n	mean ± SD	%	p
	**Age at puberty onset, years**		134	6.63 ± 1.36		359	6.67 ± 1.39		0.89
		≤ 6 years	32	-	23.9	82	-	22.8	
**Initial evaluation**	**Age, years**		134	7.54 ± 1.41		359	7.55 ± 1.49		0.94
	**Tanner stage of breast development**		134	2.58 ± 0.56		359	2.56 ± 1.59		0.91
	**Tanner stage of pubic hair development**		134	1.99 ± 0.91		357	1.99 ± 0.93		0.61
	**Bone age advance, years**		131	1.50 ± 1.30		345	1.17 ± 1.22		**0.03**
		≥ 2 years	50	-	38.2	98	-	28.4	
	**Growth rate the year before onset, SDS**		127	2.36 ± 2.35		318	2.21 ± 2.24		0.25
		≥ 2 SDS	63	-	49.6	143	-	45.0	
	**BMI, SDS**		134	1.47 ± 1.69		359	1.19 ± 1.54		**0.03**
		≥ 2 SDS	40	-	29.9	95	-	26.5	
	**Height, SDS**		134	2.11 ± 1.24		359	2.04 ± 1.33		0.74
	**Target height, cm**		132	161.7 ± 4.91		329	163.05 ± 5.37		**0.004**
	**Predicted adult height, cm**		122	161.9 ± 7.98		321	163.29 ± 7.54		0.14
	**LH/FSH peaks ratio**		134	0.91 ± 1.14		359	0.99 ± 1.14		0.22
		≥ 0.66	75	-	56.0	162	-	45.1	
	**Estradiol, pg/mL**		130	19.9 ± 16.29		349	14.22 ± 16.98		**<0.01**
		≥ 15 pg/mL	63	-	48.5	85	-	24.3	
	**Neuroradiological evaluation**		128	-	97.7	292	-	84.4	
**Patients treated**			79	-	58.9	117	-	32.6	

MWUt: Mann-Whitney U-test

## Methods

Familial history of early puberty was defined as the mothers undergoing menarche (data available in 124 mothers) before the age of 11 years and/or the presence of an early onset of puberty in the father (pubic hair development before 11 years), brother, sister or grandparents (48 patients in total) [19].

The initial evaluation included the following data: height of each parent, age at the onset of puberty, height chart, growth rate, weight, pubertal stage, BA, evaluation of the hypothalamic-pituitary-ovarian axis by measuring basal and GnRH (100 μg/m^2^)-stimulated luteinising hormone (LH) and follicle-stimulating hormone (FSH) peaks and the plasma concentrations of estradiol. For GnRH stimulation test, we used Relefact and the plasma samples were collected 0, 30, 60 and 90 min after the injection. Plasma LH, FSH and estradiol concentrations were measured with various radioimmunoassays during the study period. Each new assay for a given hormone was cross-correlated with the previous method to ensure that the results for a given parameter were comparable throughout the study period. LH and FSH concentrations were measured by two-site monoclonal immunoradiometric assays (LH-Coatria and FSH-Coatria; bioMerieux, SA, Marcy-l’Etoile, France). Estradiol was extracted with ether and measured by radioimmunoassay (Estradiol-2; Sorin Biomedica, Antony, France).

Height, growth rate and body mass index (BMI, weight in kg/height in m^2^) were expressed as SDS for chronological age [20,21]. The pubertal stage was rated [22]. The BA was assessed by R. Brauner according to the Greulich and Pyle method [23] for all patients. The TH was calculated based on the parental heights [24]. The predicted AH was calculated [25] except in 12 cases (7 treated and 5 untreated) whose predicted AH could not be calculated because they had a BA of less than 7 years; we used the column “advanced” when the bone age advance was greater than one year. The duration of puberty was calculated as the time between the onset of puberty and first menstruation.

The patients were assigned to one of two groups: treated patients (n = 78, 58.2%) were given GnRHa and untreated patients (n = 56) were followed without treatment. The criteria for treatment were a predicted AH <155 cm at initial evaluation (n = 18) or at the evaluations performed every 4 months (n = 27); an LH/FSH peaks ratio >0.66 (n = 50); and/or a plasma estradiol concentration >15 pg/mL (n = 45). Treatment (D-Trp-GnRH, 3.75 mg, i.m., every 24–26 days; half doses in patients weighing < 20 kg) was continued for at least 2 years. Oral cyproterone acetate (12.5 mg twice a day) was given for 15 days before and 1 month after the first GnRHa injection to prevent the formation of ovarian cysts in response to the initial gonadotropin surge. The regression of the breast development after 3 months of treatment was systematically verified. Plasma estradiol concentration was measured in the cases without breast regression; for all these cases, it was prepubertal (<15 pg/mL). Thereafter, the patients were seen every 6 months after the start of therapy for clinical evaluation and measurements of height and BA before deciding to stop therapy. Therapy was stopped when the BA was approximately 12–12.5 years except for 19 girls whose BA was 8–11.8 years. In these girls, the decision to stop the therapy was made with the parents on a case-by-case basis because the chronological age or height was judged to be sufficient for an increase in estradiol secretion or, more frequently, because of a slow growth rate (<3 to 4 cm) over the previous year.

Untreated patients (n = 56) were followed without treatment because their predicted AH was >155 cm in all but 5 cases (147.2–154.5 cm). Four of these 5 had low LH/FSH peaks ratios and plasma estradiol concentrations, and all 5 were older than 6.5 years. Untreated patients were followed every 4 months until they reached an acceptable predicted AH for clinical and BA evaluation, if indicated.

### Analysis

Groups were compared with a Mann-Whitney U-test (MWUt). Correlations were calculated according to Pearson’s definition, and proportions were compared with a chi square test.

Data analysis was performed using multiple linear regression models. We restricted our search to models using only variables that were known at the initial evaluation. We then reduced this set of variables by looking for small subsets that allow for high-quality models.

The evaluation of each model (that is, of each subset of variables) was computed using a classical cross-validation procedure, as follows: 80% of the dataset is randomly and uniformly picked as training data to compute a model, and the remaining 20% is used to evaluate this model. This is repeated 100 times, and the average performance of those 100 models is retained. If a model is sufficient, according to this procedure, it is re-computed for the whole dataset. This procedure simulates the performance of a model when used on new data that are consistent with the currently available data [26].

Data were expressed as means±SD.

## Results

### Comparison of the groups at initial evaluation

Treated patients were younger at the onset of puberty, but the intervals between the onset of puberty and the evaluation were similar in the 2 groups (0.94±0.66 years in treated and 0.87±0.61 years in untreated) (Table 2). Breast development was at Tanner’s stage 2 in 30.8% of treated patients, and stages 3 and 4 in 69.2%, whereas these percentages were 64.3% and 35.7%, respectively, for untreated patients (p<0.001). Treated girls had significantly greater BA advance, LH/FSH peaks ratios, plasma estradiol concentrations, and lower predicted AH than did the untreated girls. THs were similar in both groups.

**Table 2:** Characteristics of 134 girls with idiopathic CPP.

			All patients			Treated patients			Untreated patients			MWUt
		Remarkable values	n	mean±SD	%	n	mean±SD	%	n	mean±SD	%	p
	**Age at puberty onset, years**		134	6.63±1.36		78	6.42±1.53		56	6.93±1.01		**0.04**
		≤6 years	32	-	23.9	22	-	28.2	10	-	17.9	
**Initial evaluation**	**Age, years**		134	7.54±1.41		78	7.36±1.59		56	7.8±1.08		0.22
	**Tanner stage of breast development**		134	2.58±0.56		78	2.72±0.51		56	2.39±0.57		**0.0004**
	**Tanner stage of pubic hair development**		134	1.99±0.91		78	1.94±0.87		56	2.07±0.97		0.51
	**Bone age advance, years**		131	1.50±1.30		77	1.77±1.36		54	1.13±1.13		**0.004**
		≥2 years	50	-	38.2	34	-	44.2	16	-	29.6	
	**Growth rate the year before onset, SDS**		127	2.36±2.35		75	2.43±2.36		52	2.25±2.35		0.34
		≥2 SDS	63	-	49.6	40	-	53.3	23	-	44.2	
	**BMI, SDS**		134	1.47±1.69		78	1.38±1.63		56	1.6±1.78		0.78
		≥2 SDS	40	-	29.9	22	-	28.2	18		32.1	
	**LH/FSH peaks ratio**		134	0.91±1.14		78	1.14±1.23		56	0.59±0.92		**<0.0001**
		≥0.66	75	-	56.0	50	-	64.1	15	-	26.8	
	**Estradiol, pg/mL**		130	19.9±16.29		75	23.79±18.52		55	14.82±10.82		**0.0008**
		≥15 pg/mL	63	-	48.5	45	-	60.0	18	-	32.7	
**Evolution**	**Duration of puberty, years**		129	4.66±1.95		73	5.44±1.86		56	3.64±1.57		**<0.0001**
	**Age at 1st menstruation, years**		129	11.29±1.24		73	11.85±1.02		56	10.57±1.12		**<0.0001**
		≤11 years	64	-	49.6	21	-	28.8	43	-	76.8	
**Growth evolution**	**Height at initial evaluation, SDS**		134	2.11±1.24		78	1.95±1.37		56	2.35±1.0		0.09
	**Adult height, cm**		134	162.1±5.61		78	160.9±5.7		56	163.8±5.0		**0.003**
	**Target height, cm**		132	161.7±4.91		77	161.5±5.38		55	162.0±4.20		0.73
	**Predicted adult height, cm**		122	161.9±7.98		71	159.5±7.63		51	165.2±7.28		**0.0001**
	**Adult—target heights, cm**		132	0.38±5.1		77	-0.66±5.25		55	1.83±4.53		**0.009**
	**Adult—predicted heights, cm**		122	-0.04±6.81		71	0.92±7.26		55	-1.39±5.94		**0.03**

MWUt: Mann-Whitney U-test

### Treated group

The time between the onset of puberty and treatment was 1.42±0.9 (0.2–7.8) years. The time between the initial evaluation and treatment was 0.5±0.8 years. Treatment was initiated at 7.9±1.6 years and was stopped at 10.7±0.8 years. During treatment (2.8±1.4 years), the BA increased from 9.8±1.7 to 12.0±0.9 years. The height gain between the end of treatment and AH was 12.3±4.9 (0 to 29) cm. The growth rate during treatment was 5.7±1.4 cm/year. The time between the end of treatment and the first menstruation was 1.3±1.0 (0.1 to 3.5) years. The 19 girls whose treatment was stopped before reaching a BA of 12 years had characteristics that were not different from those of the other treated patients.

The AH was similar to the AH predicted before treatment and to the TH. The AH was positively correlated with the predicted AH (r = 0.43, p<0.0001), TH (r = 0.55, p<0.0001) and height SDS at initial evaluation (r = 0.59, p<0.0001). Most of the treated girls reached their TH with ±1 SD (n = 58, 74.4%), and only 12 (15.4%) had an AH lower than their TH by more than 1 SD (5.6 cm).

The AH was at -2 SD (152 cm) in 5 patients and was below this in 2 patients (Table 3). Three had BMI over 2 SD and one below -2 SD. This girl (case 1) began breast development at 5.5 years and was treated at 10.2 years because a very low evolution was followed by a rapid progression. The TH was below -1 SD in 5 patients, and the predicted AH was below—1 SD in all but case 3 who had shorter AH (147 cm) and greater height loss compared to the predicted AH in this population. In this girl, MRI identified a Rathke cyst of 7.8 mm, and the images did not progress on a second MRI performed 6 months later, leading to the conclusion that this was a variant of the normal.

**Table 3:** Characteristics of the 8 girls with adult height ≤152 cm (- 2 SD) after idiopathic CPP.

		1	2	3	4	5	6	7	8
**Initial evaluation**	**Age at puberty onset, years**	5.5	6.8	7	7	7.1	7.8	8	7.8
	**Tanner stage**	B2P2	B3P3	B3P1	B3P1	B3P3	B3P3	B3P2	B2P3
	**Bone age advance, years**	-0.4	2.9	-1.3	2.2	2.6	1.4	0	2.2
	**Growth rate the year before onset, SDS**	0.1	0.8	1.4	0.4	5.5	1.1	2.8	1.1
	**BMI, SDS**	-2.2	1.7	0.2	2.1	3.2	2.9	-0.6	3.1
	**LH/FSH peaks ratio**	0.34	1.11	2.33	1	3.95	1.58	1.99	1.03
	**Estradiol, pg/mL**	25	70	2	10	NA	9	4	10
**Treatment**		yes	Yes	yes	yes	yes	yes	yes	No
	**Age at treatment onset, years**	10.2	7.7	7.8	8.3	8.5	9.1	9.1	-
	**Treatment duration, years**	2.1	3.3	2.6	3	2.5	2	2.2	-
	**Height gain during treatment, cm**	10.3	16.1	12	14	10.7	8.3	11.5	-
	**Growth rate during treatment, cm/year**	4.9	4.9	4.6	4.7	4.3	4.2	5.2	-
	**Bone age at treatment end, years**	11.5	12.5	10	12	13	12.5	12	-
**Evolution**	**Duration of puberty, years**	7.4	5.5	3.9	4.7	4.9	4.7	NA	1.6
	**Age at 1st menstruation, years**	12.9	12.3	10.9	11.7	12	12.5	NA	9.4
**Growth evolution**	**Height at initial evaluation, SDS**	-2	1	0.9	0	1.7	0.2	-0.1	1.2
	**Adult height, cm/SDS**	152/-2	152/-2	147/-2.9	152/-2	152/-2	150.5/-2.3	152/-2	150/-2.4
	**Target height, cm/SDS**	162/-0.2	149.5/-2.5	156/-1.3	159.5/-0.7	153.5/-1.8	153.5/-1.8	153.5/-1.8	156/-1.3
	**Predicted adult height, cm/SDS**	147.2/-2.9	147.5/-2.8	169.4/1.1	148.6/-2.6	152.1/-2	152.7/-1.9	155.9/-1.3	153.6/-1.7
	**Calculated AH, cm/SDS**	154.2/-1.6	154.1/-1.6	154.3/-1.6	156.4/-1.2	152.1/-2.0	153.1/-1.8	151.6/-2.1	157.5/-1.0

### Untreated group

The AH was similar to the AH predicted at the initial evaluation but was greater than the TH (p = 0.01). The AH was positively correlated with the predicted AH (r = 0.59, p<0.0001), TH (r = 0.53, p<0.0001) and height SDS at initial evaluation (r = 0.53, p<0.0001). Most of the untreated girls reached their TH with ±1 SD (n = 43, 76.8%), and only 2 (3.5%) had an AH lower than their TH by more than 1 SD, including the only one that had an AH below -2 SD (case 8, Table 3).

First menstruation occurred before 10 years in 16 (28.6%, 2/53 in their mothers) and before 11 years in 33 (58.9%, 8/53 in their mothers). The time between onset of puberty and first menstruation was 3.6±1.5 years (0.9–7.8 years). In only 9 girls, this time was less than 2 years. Among these 9 girls, all were over 7 years old at puberty onset, 4 had familial history of CPP with mother’s first menstruation before 11 years, 8 had a LH/FSH peaks ratio over 0.66 and 4/5 evaluated had pubertal uterus length greater than 35 mm.

**Fig 2:**
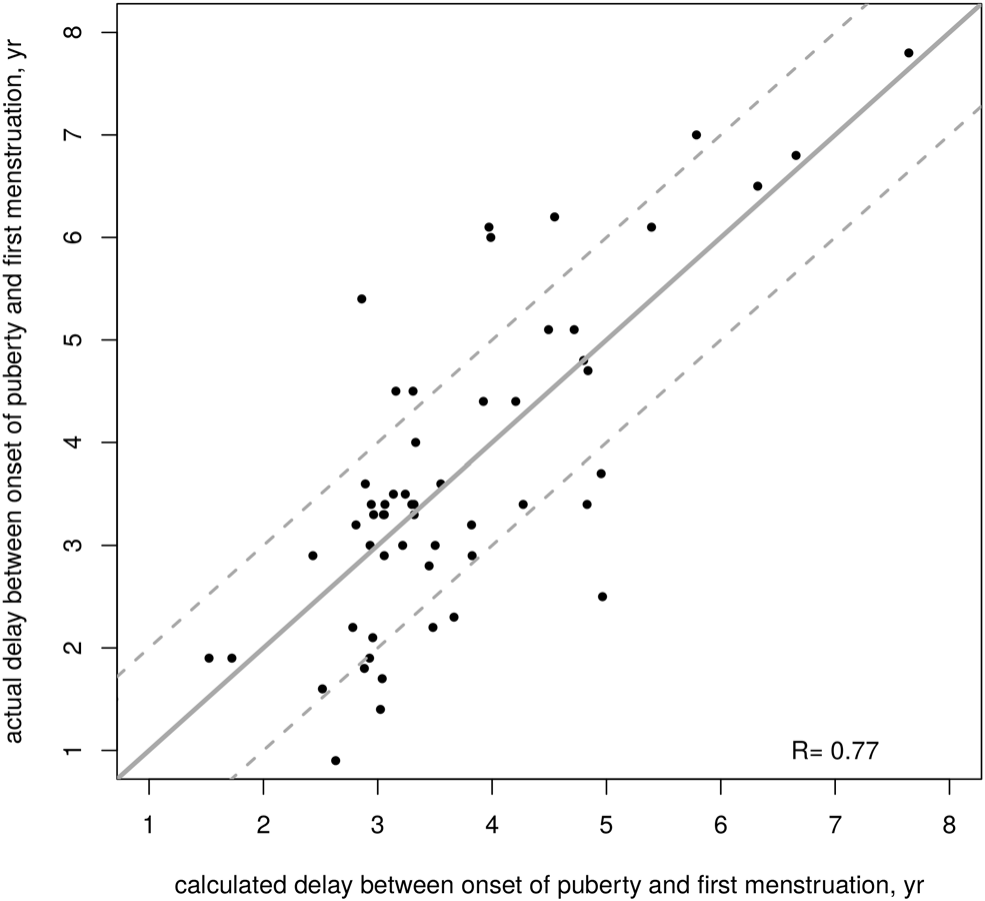
Interval time (years) between the onset of puberty and first menstruation in 56 girls with untreated idiopathic CPP using the formula from http://www.kamick.org/lemaire/med/girls-cpp15.html. Plain line represents the reference perfect prediction (calculated = actual), and dotted lines represent ± year from that value.

The time between onset of puberty and first menstruation can be estimated using the following formula (Fig. 2): 10.9–0.57 (LH/FSH peaks ratio).

This formula is available online at http://www.kamick.org/lemaire/med/girls-cpp15.html.

The model makes an average absolute error of 0.75 year (0.82 year in cross-validation); it overestimates the actual interval by more than 1 year for 10 girls (17.9%) and underestimates it by more than 1 year for 7 girls (12.5%).

### Model of prediction of the AH

**Fig 3:**
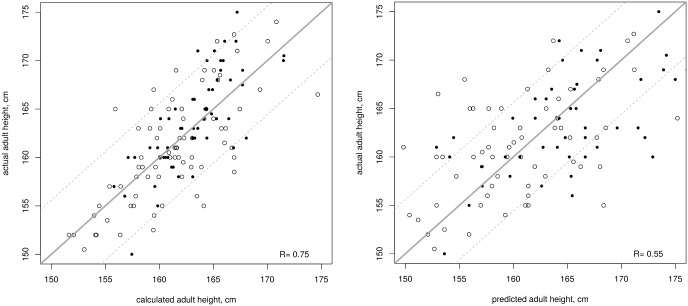
Correlation between actual adult height and calculated adult height (left) or adult height predicted by the Bayley Pinneau method [25] (right) in 78 treated (empty circles) and 56 untreated girls (plain circles) with idiopathic CPP using the formula from http://www.kamick.org/lemaire/med/girls-cpp15.html. Plain line represents the reference perfect prediction (calculated = actual), and dotted lines represent ± 1 SD from that value.

Models were first established separately for the treated and untreated girls, but they happened to be very similar and output similar predictions for the whole group. Thus, we established a model for the whole population using the following formula (Fig. 3):

AH calculated (SD) = 0.39 (height at initial evaluation, SD) + 0.41 (target height, SD)– 0.33 (LH/FSH peaks ratio)- 0.65.

AH calculated (cm) = 2.21 (height at initial evaluation, SD) + 2.32 (target height, SD)– 1.83 (LH/FSH peaks ratio) + 159.68.

This formula is available online at http://www.kamick.org/lemaire/med/girls-cpp15.html.

The data required are the age (years) and height (cm) at the initial evaluation, the fathers’ and mothers’ heights (cm), and LH and FSH peaks. The model makes an average absolute error of 0.52 SD (2.91 cm) on new data. For the currently available data, the average absolute error is 0.52±0.42 SD (2.91±2.35 cm), and the worst errors are -1.63 SD (-9.13 cm) and 1.62 SD (9.07 cm). For treated girls, the average absolute error is 0.56±0.44 SD (3.14±2.46 cm), and the worst errors are -1.63 SD (-9.13 cm) and 1.62 SD (9.07 cm). For untreated girls, the average absolute error is 0.47±0.38 SD (2.58±2.13 cm), and the worst errors are -1.33 SD (-7.45 cm) and 1.39 SD (7.78 cm).

The calculated AH (162.12±4.24 cm) and the actual AH are highly correlated (r = 0.75, Fig. 3). The actual AH was lower than the calculated AH by more than 1 SD (5.6 cm) in 11 girls (8 treated and 3 untreated), and greater in 10 girls (6 treated and 4 untreated).

In comparison, the predicted AH obtained using the method of Bayley and Pinneau (161.9±7.98 cm) is less correlated with the actual AH than the calculated AH (r = 0.55) and makes significantly larger errors: the average absolute error is 0.91±0.80 SD (5.01±4.48 cm) and the worst errors are -4 SD (-22.4 cm) and 3.76 SD (21.1 cm).

## Discussion

We established formulae that can be used at an initial evaluation to predict both the AH in a given girl with CPP and the interval time between the onset of puberty and the first menstruation after spontaneous puberty. According to the formula, the actual AH was lower than the calculated AH by more than one SD (5.6 cm) in 11 girls (8.0%) (10.2% of the treated and 5.3% of the untreated girls).

### Comparison of the groups at initial evaluation

The treated girls were younger at the onset of puberty than were the untreated girls; the interval between the onset of puberty and the initial evaluation was similar in both groups, as was the TH. Treated girls had significantly greater BA advance, LH peaks, LH/FSH peak ratios, plasma estradiol concentrations, and lower predicted AH than did the untreated girls. These findings are not surprising because the characteristics that differentiate the groups correspond to the criteria that we chose for the indications of GnRHa treatment.

### Formulae for prediction

The formula that can be used at the initial evaluation to predict AH includes age and height at the initial evaluation (to calculate the SD), the height of each parent, and the LH/FSH peaks ratio, while the formula of the prediction of the interval time between the onset of puberty and first menstruation after spontaneous growth includes only the LH/FSH peaks ratio. In two previous studies, we used mathematical models to predict the difference between the AH and TH in girls with idiopathic CPP [13] and to predict AH in girls with advanced puberty [27]. In these two models, as in the present study, greater TH and actual height at the initial evaluation were associated with greater AH, in both the treated and untreated CPP groups and advanced puberty. The LH/FSH peaks ratio was included in our previous model of untreated girls with CPP and was selected by the construction of the models, whereas the other variables (listed in the Table of characteristics) were not. This is not surprising because TH is a major determinant of AH in children with and without growth diseases. The height at the initial evaluation integrates the height level before and after the acceleration of the growth rate. In girls with CPP, this acceleration is due to the estradiol secretion, but the growth rates of CPP patients are independent of the plasma estradiol concentrations. However, these concentrations are correlated with the bone age advance [28], and height at the start of treatment appeared to be the most important factor positively influencing AH (r = 0.75) [11].

It has been shown that the LH/FSH peak ratio after the GnRH test <0.66 is the gold standard of the maturation of the hypothalamic-pituitary-ovarian axis [29]. The LH/FSH peak ratio is significantly increased with the number of signs of puberty associated with breast development at the evaluation [15]. We have previously shown that this ratio is significantly correlated with anterior pituitary height on MRI [30]. In the present study, among the 9 girls with interval time between onset of puberty and first menstruation less than 2 years after spontaneous puberty, 8 (among 15 in the whole untreated group) had a LH/FSH peaks ratio over 0.66. In the study including the 493 girls with CPP, we found that the girls with a familial history of early puberty had a significantly greater frequency of pubertal LH/FSH peaks ratio [15]. This result suggests that the maturation of the hypothalamic-pituitary-ovarian axis depends on genetic factors, which impacts the type of the evolution of CPP.

The formulae in [13] and the current study are coherent. The calculated AH in both studies are positively correlated (r = 0.81 for treated girls; r = 0.96 for untreated girls), as is the interval between the onset of puberty and first menstruation (r = 0.88). The average absolute error is clearly not negligible. However, compared to the Bayley and Pinneau method [25], the formula gives a predicted AH which better correlates with the actual AH and makes significantly lower errors both for the average absolute error and for the worst errors. Furthermore, to apply the proposed formulae does not imply prohibitive costs, since the required pieces of data are only basic clinical measures, except the LH/FSH peak ratio.

### Therapeutic indications

**Fig 4:**
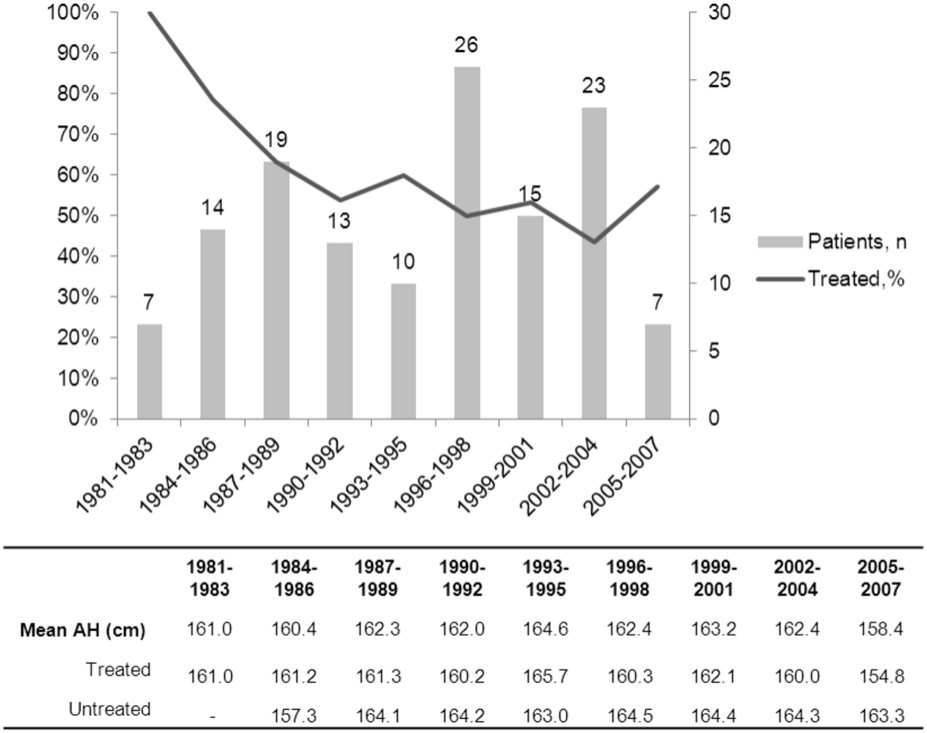
Evolution of consultations and treated girls rates over 26 years in the 134 girls with CPP of the present study. Plain line represents the average of the proportion of treated girls over 7 years (current year, 3 years before and 3 years after).

Considering the whole population of CPP, which has been studied for 32 years [15], and the present study, which is limited to those aged over 14 years (26 years), the percentage of the girls with CPP treated with GnRHa varies from year to year, with a clear decreasing trend: this rate of treatment was approximately 70% in the early 1980s and has decreased to approximately 50% in the late 1990s (Fig. 4). The mean AH of the global population and of the treated and untreated groups has not significantly varied over this time. The characteristics at the initial evaluation and the evolution of both the present population and those whose AH we reported in 1994 [2] and in 2002 [14] did not change, including the percentage of those aged less than 6 years. The decrease in the percentage of girls treated is due to our decision to non-stop the secretion of the estradiol. However, this decision has important psychological implications. It is difficult for a girl aged less than 10 years to have pubertal development, mainly menstruations. The criteria of Tanner stage breast development had not been included in the decision to treat, but the inclusion of plasma estradiol concentration in the criteria is associated with significantly greater breast development in the treated group.

### Study limitations

This study has several limitations. It is retrospective, but it includes a large number of treated and untreated patients who were followed by the same physician. The girls who were excluded because of the change in their address or no answer (Fig. 1 and Table 1) may introduce bias. We postulate that the similarity of these girls to the girls who were included, with regard to the variables analyzed (except for the BA advance and plasma estradiol concentrations), limits this bias. The decision to stop treatment was based on a BA at approximately 12–12.5 years, which is associated with better growth until AH [2]. However, 19 girls had lower BAs when the decision to stop the therapy was made because their chronological age or their height was judged sufficient for an increase in estradiol secretion or, more frequently, because their growth rate had been slow over the previous year. These 19 girls had characteristics similar to those of the other treated girls, including their age at first menstruation and their AH. The AHs of a few girls were collected from health records held by their pediatricians. The reported parental height is less accurate than the measured heights. The major limitation in our study is the lack of validation of the formulae on a separate population.

## Conclusion

This study provides a useful and ready-to-use formula for AH prediction, with a significant error (>1 SD) limited to 15.9% of the cases. Internal validation was reached, but external validation is necessary. However, the data included in the formula (heights and LH/FSH peaks ratios) are not exposed to errors of interpretation. The age at first menstruation can also be predicted. However, the evolution of the estrogenisation varies over time, as does the response to the GnRH test. This explains the difficulty of using a randomized protocol for deciding treatment.

The similarity of the formulae obtained for the treated girls, the untreated girls, and the whole group, suggests that the GnRHa treatment had no significant effect on AH. However, the criteria used to select (generally the LH/FSH peaks ratio) a treatment suggest that this treatment prevents the deterioration of AH in the cases with a rapidly evolving form of CPP. Girls with a slowly evolving form of CPP may progress into a rapidly evolving form, leading to a secondary decision to give GnRHa, which emphasizes the necessity of following untreated patients until they reach a sufficient height.
